# Droplet-digital PCR reveals frequent mutations in *TERT* promoter region in breast fibroadenomas and phyllodes tumours, irrespective of the presence of *MED12* mutations

**DOI:** 10.1038/s41416-020-01109-8

**Published:** 2020-10-13

**Authors:** Kazutaka Otsuji, Takeshi Sasaki, Masahiko Tanabe, Yasuyuki Seto

**Affiliations:** 1 Department of Breast and Endocrine Surgery, Graduate School of Medicine, The University of Tokyo, Tokyo, Japan; 2 Department of Next-Generation Pathology Information and Networking, Faculty of Medicine, The University of Tokyo, Tokyo, Japan; 3 Department of Gastrointestinal Surgery, Graduate School of Medicine, The University of Tokyo, Tokyo, Japan

**Keywords:** Breast cancer, Cancer genomics, Cancer genetics, Cancer genetics

## Abstract

**Background:**

Breast fibroadenoma (FA) and phyllodes tumour (PT) often have variations of gene mediator complex subunit 12 (*MED12*) and mutations in the telomerase reverse transcriptase promoter region (*TERTp*). *TERTp* mutation is usually tested by Sanger sequencing. In this study, we compared Sanger sequencing and droplet-digital PCR (ddPCR) to measure *TERTp* mutations in FA and PT samples.

**Methods:**

FA and PT samples were collected from 82 patients who underwent surgery at our institution from 2005 to 2016. *MED12* mutations for all cases and *TERTp* mutations for 17 tumours were detected by Sanger sequencing. ddPCR was performed to analyse *TERTp* mutation in all cases.

**Results:**

A total of 75 samples were eligible for analysis. Sanger sequencing detected *MED12* mutations in 19/44 FA (42%) and 21/31 PT (68%). Among 17 Sanger sequencing-tested samples, 2/17 (12%) were *TERTp* mutation-positive. In ddPCR analyses, a significantly greater percentage of PT (19/31, 61%) was *TERTp* mutation-positive than was FA (13/44, 30%; *P* = 0.0046). The mutation positivity of *TERTp* and *MED12* did not correlate, in either FA or PT.

**Conclusions:**

ddPCR was more sensitive for detecting *TERTp* mutation than Sanger sequencing, being able to elucidate tumorigenesis in FA and PT.

## Background

Breast fibroepithelial tumours, which include fibroadenoma (FA) and phyllodes tumour (PT), are characterised by the biphasic proliferation of both epithelial and stromal components.^1–5^ FA is common benign tumour that is often observed in young women. It expresses oestrogen receptor (ER)-α in epithelium and ERβ in stromal components, and is known to be hormone dependent.^6,7^ Small FA (<3 cm) is usually followed up without resection, and 16–37% of FA cases reportedly regress or completely resolve spontaneously.^8–10^ PT is much less common than FA; it comprises only 2–3% of fibroepithelial breast tumours and accounts for <1% of all breast tumours. PT is histologically classified as benign, borderline or malignant type. Resection is generally recommended for PT because it often grows rapidly and can potentially become malignant. Occasionally, PT enlarges to huge sizes that require total mastectomy, and malignant PT has high risks of both local recurrences and distant metastasis compared with the other types of PT.^1–3,10–15^

FA and PT, especially when they remain relatively small, are morphologically so similar that differentiating them histologically is sometimes difficult. Some studies have suggested that FA could potentially progress to PT, especially FA with monoclonal stromal components; however, the mechanisms underlying their initiation and progression are unclear.^10,11,16,17^ Although FA and PT have been genomically analysed,^18–21^ little had been known about their genetic abnormalities. However, since next-generation sequencing (NGS) became widely used in research, several genetical alterations have been revealed in both tumours. The discovery of highly recurrent Mediator complex subunit 12 (*MED12*) somatic mutations in breast FA was surprising; almost nothing had previously been known about these mutations.^22^ As for PT, in addition to *MED12*,^10–13,23–27^ the telomerase reverse transcriptase (*TERT*) promoter has been shown to have repeated mutations in these tumours.^28–34^

In 2014, Lim et al. first found highly frequent *MED12* exon 2 mutations in FA (58/98, 59%) using exome analysis.^22^ The *MED12* gene, located on the X chromosome, encodes MED12 protein, a member of the multiprotein mediator complex that regulates transcription of all RNA polymerase II-dependent genes.^35^ Reportedly, up to 60% of FA, 80% of benign and borderline PT and 40% of malignant PT harbours somatic mutations in exon 2 of the *MED12* gene,^10–13,22–27^ which suggests that FA and PT have much more in common in their origin or development than previously thought. However, the underlying mechanisms, and how this mutation generates or induces the progression of FA and PT, are unknown.

Since the discovery of frequent *MED12* mutations in FA and PT, next-generation sequencing has been used to search for other gene mutations in these tumours. Many mutations found in PT, such as in *RARA*, *EGFR*, *RB1* or *TP53*, are very uncommon compared with *MED12* mutations,^12,21,27–30^ whereas mutations in the *TERT* promoter region (*TERTp*) are reportedly more frequent: 0–7% in FA, and 27–70.6% in PT.^29–34^ *TERTp* is considered to be a critical regulatory element for telomerase expression.^35,36^ Hotspots for mutations in this region in PT and FA are reported to be c.−146 C > T (C250T) and c.−124 C > T (C228T),^29,31–34^ which are concordant with those in other tumours, including central nervous system tumours, thyroid cancers, bladder cancers and skin melanoma.^36,37^ Previous studies found correlations between *TERTp* mutations (*TERTp*^*Mut*^) and *MED12* mutations (*MED12*^*Mut*^),^28–31^ which suggests that these mutations interact, especially in the development of PT.

Recently, McEvoy et al. showed that droplet-digital polymerase chain reaction (ddPCR) is very sensitive in detecting *TERTp*^*Mut*^ in melanoma compared with pyrosequencing or Sanger sequencing (SS).^38,39^ Digital PCR was developed to yield absolute measures for nucleic acid concentrations by a combination of limiting dilution, end-point PCR and Poisson statistics.^40^ ddPCR is a newer, more precise and less subjective assay to quantify DNA amplification, based on water–oil emulsion droplet technology. In ddPCR, a sample is fractionated into 20,000 droplets, and PCR amplification of the template molecules occurs in each individual droplet. ddPCR has also been shown to obtain high levels of partitioning at a low cost.^41,42^

*TERTp*^*Mut*^ was previously evaluated with ddPCR, but only in melanoma.^38,39^ We found no reports of studies that evaluated mutations in breast fibroepithelial lesions using ddPCR. In this study, we used ddPCR to measure *TERTp*^*Mut*^ in formalin-fixed, paraffin-embedded (FFPE) samples of resected FA or PT, and compared the results with those from conventional SS. We also analysed the relationships among *TERTp*^*Mut*^ and *MED12*^*Mut*^ status and histopathological characteristics in FA and PT.

## Methods

### Tissue samples

We collected FFPE samples of 54 FA and 31 PT from 82 patients who underwent surgery at the University of Tokyo Hospital from 2005 to 2016. Three patients had two tumours excised at different times. All samples were diagnosed by two expert pathologists. FA was classified as intracanalicular type, pericanalicular type, mastopathic type, organoid type,^43,44^ complex fibroadenoma^45^ or juvenile fibroadenoma;^46^ PT was subclassified according to the WHO classification as benign, borderline or malignant lesions.^2^ All patients consented to the use of their stored tumour tissue. This study protocol was approved by the ethics committee at the University of Tokyo Hospital, Tokyo, Japan.

### DNA extraction

For each specimen, two or three 10-µm FFPE sections were cut from a single representative block per case. Macrodissection was performed with a scalpel as needed to adjust the tumour content to be visually more than 20%. Microdissection was not performed. DNA was isolated using a GeneRead DNA FFPE Kit (Qiagen, Hilden, Germany), in accordance with the manufacturer’s instructions. Purified DNA was quantified using a NanoDrop 2000 spectrophotometer (Thermo Scientific, Wilmington, DE, USA), with 0.5–14.0 µg of DNA recovered per section.

### Sanger sequencing to detect MED12 mutation

FA and PT were analysed for mutations in exon 2 of *MED12* by SS for all samples. Exon 2 was amplified with the following primers: (exon 2 forward) 5′-AACTAAACGCCGCTTTCCTG-3′, (exon 2 reverse) 5′-TTCCTTCAGCCTGGCAGAG-3′^10,47^ (Supplementary Table S1). The PCR products were purified using agarose gel electrophoresis, labelled with Big Dye Terminator (Applied Biosystems, Foster City, CA, USA) with bidirectional primers and subjected to 3130 × l Genetic Analyzer (Applied Biosystems, Foster City, CA, USA) in accordance with standard protocols. The Catalogue of Somatic Mutations in Cancer (COSMIC) database^48^ was used to identify already-known somatic mutations and mutation types.

### Sanger sequencing for detecting TERTp mutations

*TERTp* mutations (C228T and C250T) were analysed in FA and PT with the same extracted DNA as used for the *MED12*^*Mut*^ analysis. We performed SS on 17 samples, which had been surgically resected in 2015 and 2016. *TERTp* was amplified with the following primers: (promoter forward) 5′-AGCGCTGCCTGAAACTCG-3′, (promoter reverse) 5′-CCTGCCCCTTCACCTTCCAG-3′^31,34^ (Supplementary Table S1). Q-Solution (Qiagen) was added to the reaction to amplify *TERTp* by PCR, in accordance with previous reports.^38,39^ The SS method was the same as that for the *MED12*^*Mut*^ analysis described above.

### ddPCR for detecting TERTp mutations

Analysis of *TERTp* mutations (C228T and C250T) with ddPCR was performed for all of the samples, using a previously described ddPCR method.^38,39^ Primers were the same as those used in SS (Supplementary Table S1). Two probes were used to detect the mutations: one (the mutant probe) was designated as “/56-FAM/CCC + C + T + T + CCGG/3IABkFQ/” to detect both C228T and C250T mutations (as both mutations result in the same sequencing string), and the other (the wild probe) was designated as “/5HEX/CCCC + C + T + CCGG/3IABkFQ/,” to recognise the C228 locus (Supplementary Fig. S1). The probes were custom-synthesised by Integrated DNA Technologies (Coralville, IA, USA). PCR reactions were performed in 20-mL reactions that contained 10 µL of Bio-Rad 2× ddPCR Supermix for Probes (no dUTP) (Bio-Rad, Hercules, CA, USA), 250 nmol/L probe, 900 nmol/L primers, 10–290 ng of DNA and water. Reaction mixtures were partitioned into emulsions of ~20,000 droplets in oil using a QX200 Droplet Generator (Bio-Rad). The droplets were then transferred to a 96-well PCR plate, heat-sealed and placed in a thermal cycler (Bio-Rad PX1). Droplets were generated and analysed using the QX200 Droplet Digital PCR System (Bio-Rad). The thermal cycling conditions were 1 cycle at 95 °C (2.5 °C/s ramp) for 10 min, 40 cycles at 94 °C (2.5 °C/s ramp) for 30 s and at 57 °C followed by 98 °C (2.5 °C/s ramp) for 10 min. Samples were held at 4 °C until further processing. After PCR, the PCR plates were loaded on a Bio-Rad QX200 droplet reader, and ddPCR absolute quantifications of mutant and wild-type alleles were estimated by modelling them as a Poisson distribution, using Bio-Rad QuantaSoft version 1.6.6 software. Thresholds were defined based on signals from empty droplets, wild-type DNA controls and mutant-positive controls, as described in the Droplet Digital Application Guide (Bio-Rad).

### Statistical analysis

All analyses were performed using JMP Pro statistical software (ver. 14.1.0, SAS Institute, Tokyo, Japan). Fisher’s exact test or chi-squared test was used to analyse categorical data. Proportions were compared by two-sample tests. The *t* test, Mann–Whitney *U* test or Kruskal–Wallis test was used to analyse continuous variables. *P* < 0.05 was considered significant.

## Results

Among 85 FFPE tissue samples, 75 (44 FA and 31 PT) were eligible for analysis. Ten samples, including five FA samples with insufficient extracted DNA and five FA samples in which the *MED12* amplicon could not be amplified to perform SS, were excluded. We successfully performed ddPCR to detect *TERTp* mutations in all PT samples and SS for *TERTp* regions in all but one PT sample.

### Patient characteristics

Histological classifications of FA, histological grades of PT, ages and tumour sizes of each case are listed in Table 1. Among 44 FA, 18 samples were typed as intracanalicular, 6 as pericanalicular, 8 as mastopathic, 7 as organoid, 4 as complex and 1 as juvenile fibroadenoma. Among 31 PT samples, 17 were classified as benign, 9 as borderline and 5 as malignant. The FA ranged in size from 9 to 125 mm, and the PT ranged from 23 to 130 mm. The Mann–Whitney *U* test showed that patients with PT were significantly older (*P* = 0.006) and had larger tumours (*P* < 0.001) than those with FA.

**Table 1:** Clinicopathological characteristics of fibroadenomas and phyllodes tumours.

Fiboroadenomas		Phyllodes tumours		*P* value
Number of patients	43	Number of patients	29	
Number of tumours	44	Number of tumours	31	
Age (years)	38 (18–60)	Age (years)	42 (21–73)	0.006
Tumour size (mm)	27 (5–80)	Tumour size (mm)	60 (10–130)	<0.001

Classification	No. of tumours	Histological grade	No. of tumours	
Intracanalicular	18	Benign	17	
Pericanalicular	6	Borderline	9	
Mastopathic	8	Malignant	5	
Organoid	7			
Complex	4			
Juvenile	1			

Age and size are indicated as the median (range).

### MED12 mutations in FA and PT

A significantly higher percentage of PT (21/31, 68%) was *MED12*^*Mut+*^ than was FA (19/44, 42%, *P* = 0.035, two-sample test of proportions). Among FA, the intracanalicular type showed *MED12* mutations more frequently than the other types (*P* = 0.046, chi-squared test). The frequency of *MED12* mutations did not significantly differ among histological grades of PT (*P* = 0.81, chi-squared test; Supplementary Table S2).

Table 2 shows the types and locations of *MED12* mutations. All of the missense mutations have already been reported in COSMIC,^47^ but many deletion and deletion/insertion patterns were newly discovered in this study (shown in red in Table 2).

**Table 2:** Types and locations of mutation in *MED12* detected in fibroadenomas and phyllodes tumours.

Type	Mutation (cDNA level)	Amino acid	Mutated tumour number in FA	Mutated tumour number in PT
Missense	c.103 G > A	p. E35K	0	1
	c.107 T > G	p. L36P	0	1
	c.128 A > C	p. Q43P	2*	0
	c.130 G > A	p. G44S	4	6*
	c.130 G > C	p. G44R	1	0
	c.130 G > T	p. G44C	1	1
	c.131 G > A	p. G44D	2	2
	c.131 G > C	p. G44A	0	2
	c.131 G > T	p. G44V	1	4
Deletion	**c.112–144del33**	**p. A38_Q48del**	1	0
	**c.113_149del37**	p. A38Vfs*47	0	2*
	**c.114–155del42**	**p. L39_S52del**	0	1
	c.117–134del18	p. L39_G44del	1	0
	**c.118** **A** **>** **C, c.123–158del36**	**p. N40H, pK42_G53del**	1	0
	**c.122–154del33**	**p. V41_V51del**	1	0
	c.122–160del39	p. V41_G53del	1	0
	**c.125–154del30**	**p. K42_V51del**	1	0
	c.141–167del27	p. Q48_H56del	1	0
Indel	**c.100–11_136del48insC**	**Loss of splice acceptor**	0	1
	**c.137–161del25insC**	**p. N46_D54** **>** **T**	1	0
Total			19	21

cDNA changes are posted in COSMIC [47], while changes (in bold) are not.

The asterisk indicates that the tumours include reccurent cases.

*FA* fibroadenoma, *PT* phyllodes tumour.

### TERTp mutations in FA and PT

**Fig. 1:**
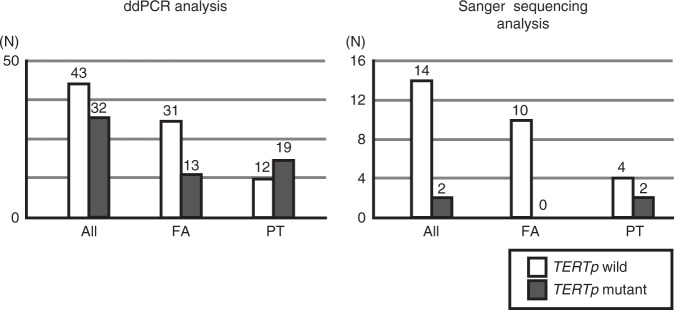
Comparison of TERTp-mutation analysis between ddPCR and Sanger sequencing. SS was performed for 16 cases to detect *TERTp*^*Mut+*^ and ddPCR was performed for all cases. SS found that 12.5% (2/16) were *TERTp*^*Mut+*^, and both the cases were PT. ddPCR analyses of all tumours found that 42.7% (32/75) were *TERTp*^*Mut+*^, a much higher rate than in SS detection. *TERTp*^*Mut+*^ was significantly more frequent in PT (19/31, 61.3%) than in FA (13/44, 29.5%; *P* = 0.009, Fisher’s exact test).

In this study, the only mutation site for *TERTp* was C228T; we saw no case of C250T. In 16 cases examined for *TERTp*^*Mut+*^ by both SS and ddPCR, SS found that 12.5% (2/16) were *TERTp*^*Mut+*^, whereas ddPCR found 37.5% (6/16; *P* = 0.037, two-sample test of proportions). Both cases determined as *TERTp*^*Mut+*^ by SS were PT; no FA case carrying *TERTp* mutations was found (Fig. 1 and Supplementary Table S3).

ddPCR analyses of all tumours found that 42.7% (32/75) were *TERTp*^*Mut+*^, which was significantly more frequent in PT (19/31; 61.3%) than in FA (13/44; 29.5%; *P* = 0.009, Fisher’s exact test, Fig. 1 and Table 3).

**Table 3:** Correlation between *TERT* promoter mutation status, analysed with ddPCR, and clinicopathological variables in fibroadenomas and phyllodes tumours.

	*TERT* promoter mutation		*P* value
	Positive	Negative	
*Tumour number*			
Total	32 (43%)	43 (57%)	
FA	13 (30%)	31 (70%)	0.009
PT	19 (61%)	12 (39%)	
*FA*			
Age (year old)	34 (19–46)	40 (18–60)	0.076
Tumour size (mm)	38 (15–73)	25 (5–70)	0.015
Classification			
Intracanalicular	4 (22%)	14 (78%)	0.51
Others	9 (35%)	17 (65%)	
*MED12* mutation			
Positive (*n*)	4 (21%)	15 (79%)	0.75
Negative (*n*)	9 (36%)	16 (64%)	
*PT*			
Age (year old)	44 (25–67)	42 (21–72)	0.57
Tumour size (mm)	50 (10–105)	49 (23–130)	1.0
Grade			
Benign (*n*)	10 (59%)	7 (41%)	0.92
Borderline (*n*)	6 (67%)	3 (33%)	
Malignant (*n*)	3 (60%)	2 (40%)	
*MED12* mutation			
Positive (*n*)	15 (71%)	6 (29%)	0.13
Negative (*n*)	4 (40%)	6 (60%)	

Age and size are indicated as the median (range).

*FA* fibroadenoma, *PT* phyllodes tumour.

**Fig. 2:**
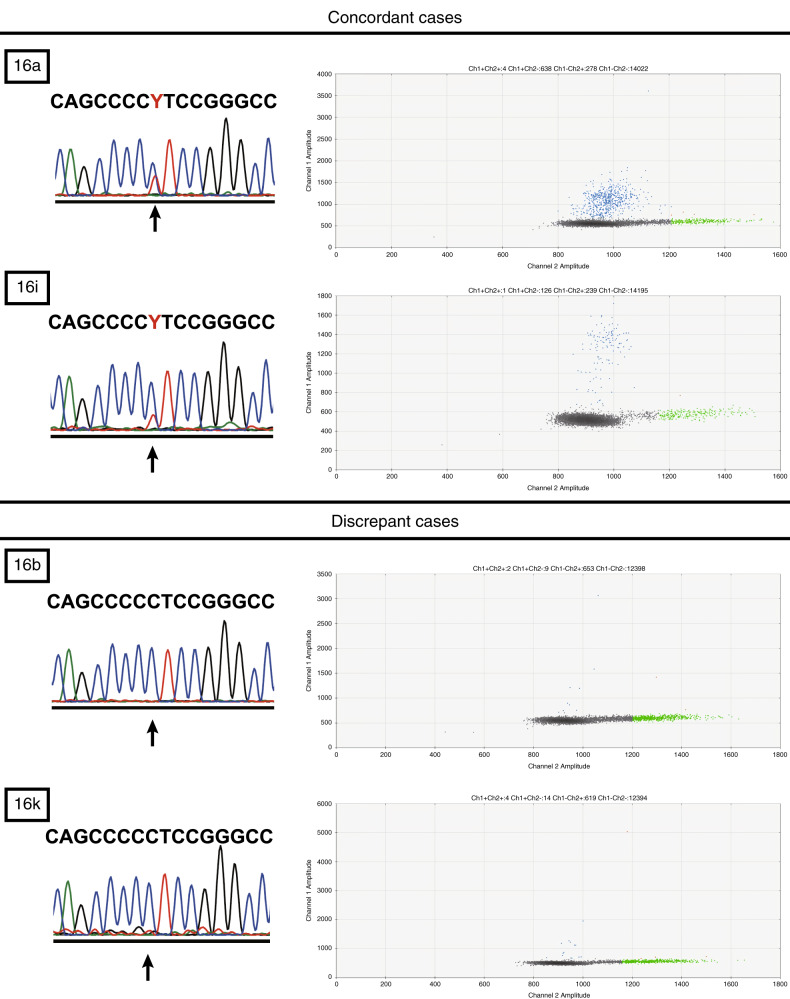
Representative traces of *TERT* promoter mutation detected by Sanger sequencing and ddPCR. Both SS and ddPCR detected C250T in Cases 16a and 16i. However, in Cases 16b and 16k, C250T was slightly positive in ddPCR analysis, but not detectable in SS.

The two samples that were found to be *TERTp*^*Mut+*^ by SS (16a and 16i) were strongly positive in ddPCR analysis, whereas those with discrepant results between SS and ddPCR (e.g. 16b and 16k) were weakly positive by ddPCR (Fig. 2).

Correlations between *TERTp-*mutation status and clinicopathological variables in FA and PT are shown in Table 3. Among FA, *TERTp*^*Mut+*^ tumours were significantly larger than *TERTp*^*Mut–*^ ones (*P* = 0.015, Mann–Whitney *U* test), but this was not the case in PT (*P* = 1.0). None of the other variables, such as patients’ age, FA classification, PT grade and *MED12-*mutation status, differed significantly between *TERTp*^*Mut+*^ and *TERTp*^*Mut−*^ tumours.

### Fractional abundance of FAs and PTs with TERTp mutation

A total of 70 FA and PT samples with *TERTp* mutation were detected by ddPCR at a fractional abundance from 0.7% to 92% (Supplementary Table S4). PT had significantly higher fractional abundance of *TERTp* mutation than FA: the mean fractional abundance in FA was 20% (range: 1.0–89%), while that in PT was 40% (range: 0.7–92%) (*P* = 0.049, Mann–Whitney *U* test). Among the cases analysed by both SS and ddPCR, fractional abundance was relatively high in the two concordant cases (28.8% and 52.3%; Supplementary Table S5). Those cases that were both *MED12-*mutation-positive with SS and *TERTp-*mutation-positive with ddPCR did not show high fractional abundance compared with the others (*P* = 0.067, *t* test; Supplementary Table S4).

### Concordance of MED12 and TERTp mutations between metachronous multiple tumours from the same individuals

In this study, three patients had metachronous multiple tumours. All three cases harboured the same *MED12* mutation in the primary and secondary lesions (Table 4). However, only one case, of malignant PT, was *TERTp*^*Mut+*^ in both primary and secondary tumours, whereas the other two cases carried this mutation only in the second tumour.

**Table 4:** *MED12* and *TERT* promoter mutations in recurrent cases.

Case	Surgical resection	Age, year old	Size, mm	Histology	Classification	*MED12* mutation	*TERTp* mutation
1	1st	28	70	FA	Complex	c.128 A > C	Wild
	2nd	30	63	FA	Organoid	c.128 A > C	C228T
2	1st	32	48	PT	Benign	c.130 G > A	Wild
	2nd	36	70	PT	Benign	c.130 G > A	C228T
3	1st	55	70	PT	Malignant	c.113_149del37	C228T
	2nd	58	10	PT	Malignant	c.113_149del37	C228T

*FA* fibroadenoma, *PT* phyllodes tumour.

## Discussion

In this study, we describe a method to detect *TERT* promoter mutations in FA and PT using ddPCR. *TERT* promoter mutation has been reported to be one of the most prevalent mutations in PT other than *MED12* mutation. ddPCR not only succeeded in detecting *TERT* promoter mutations, but also revealed this mutation to be much more frequent among breast fibroepithelial tumours, especially in FA, than was previously known. The advantages of ddPCR are its high sensitivity, quantitative measurement and low running cost. As ddPCR can detect mutations that have few patterns of single-base substitution, it is entirely appropriate for *TERTp* mutations, because only two hotspots have been reported in this region.^29,31–34^ ddPCR can distinguish mutations with a very low frequency of allele variants that SS would not have detected. Here, we show the potential high sensitivity of ddPCR in detecting *TERTp*^*Mut+*^ FA and PT, as in a previous report on melanoma by McEvoy et al.^39^

Around the mutation sites of C228T and C250T, *TERTp* region contains guanine and cytosine in high frequency. Such GC-rich regions affect the generation of secondary products during PCR, and as a consequence, inhibit DNA polymerases from copying these sites correctly. Besides, the two mutation hotspots, C228T and C250T, locate close to each other. These inconvenient factors reduce the sensitivity in sequencing analyses, including NGS. Such disadvantageous potentials remain challenging in ddPCR research; however, Corless BC et al.^49^ showed excellent sensitivity and specificity in detecting *TERTp* mutations by ddPCR. According to them, in ddPCR analysis of mutations in GC-rich regions, shorter amplicon length provides better detection sensitivity. They mentioned that DNA in FFPE samples is shortly fragmented due to the chemical reaction between formalin and nucleic acid, which helps primers bind to the target regions and generate short amplicons, leading to the efficient PCR reaction.^49^ Although FFPE samples are often considered to be unfavourable in sequencing analysis due to the low quality of nucleic acid conditions, they might be even advantageous in ddPCR analysis for *TERTp*-mutation detection.

There have been a few reports comparing the detection of genetic mutations with SS and digital PCR, including one from McEvoy et al.^39^ and another from Sho et al.^50^ McEvoy et al. examined mutations in *BRAF*, *NRAS* and *TERTp* in melanoma. According to them, the frequency of mutation detection in SS was 20% (8/40) in *BRAF*, 0% (0/40) in *NRAS* and 12.5% (5/40) in *TERTp*, whereas that with ddPCR was 55% (22/40) in *BRAF*, 10% (41/40) in *NRAS* and 37.5% (15/40) in *TERTp*.^39^ Sho et al. performed SS on resected specimens of the pancreas, and digital PCR on preoperative endoscopic ultrasound–fine-needle aspiration specimens. All 22 cases positive for mutation by SS were also determined to be mutation-positive by digital PCR, and digital PCR detected five more mutations. This report demonstrates the utility of digital PCR in cytology samples, which have a much lower tumour burden than surgical specimens.^50^ In these reports, digital PCR determined as mutation-positive for all cases in which SS detected the mutation, and this result is consistent with ours.

In this study, we used SS instead of NGS to detect *TERTp* mutations in some cases. NGS’s deep sequencing is well known to be more sensitive than Sanger sequencing, and it would be interesting to know what the results of NGS analysis would be in these samples. However, conducting NGS targeting only the *TERT* promoter region is too costly. In addition, because one of our future goals is to apply our method to clinical practice, we judged it impractical to perform additional NGS in this study from the beginning of the research.

With our *TERTp*^*Mut*^ analysis by ddPCR, the frequency of this mutation was shown with histological grade, from FA (29%) and benign PT (59%) to borderline PT (67%, Table 3). Nault et al. analysed *TERTp* mutations in cirrhotic dysplastic nodules, which are premalignant lesions of hepatocellular carcinoma (HCC), and in early and progressed HCC. They reported a strong relationship between *TERTp* mutations and hepatocarcinogenesis: the mutations were identified in 6% of low-grade dysplastic nodules, 19% of high-grade nodules, 61% of early HCC and 42% of small and progressed HCC.^51^ Our results in breast FA and PT were quite similar to their results in HCC, in that the frequency of *TERTp* mutations increased as tumours went from benign to malignant, which implies a sequential development by some FA into PT. Interestingly, our study showed a positive correlation between FA size and frequency of *TERTp* mutations (*P* = 0.015, Table 3). Some FA, especially large ones, may be genetically similar to benign PT, although morphologically distinguishable from them.

The relationship between *MED12* and *TERTp* mutations varies in previous reports.^29–31,33,34^ Piscuoglio et al.,^29^ Pareja et al.^30^ and Liu et al.^34^ reported that just 50% and slightly over of PT were *MED12*^*Mut+*^ (13/25, 5/9 and 3/6), whereas Yoshida et al.^31^ and Garcia-Dios et al.^33^ reported that almost all of them were simultaneously *MED12*^*Mut+*^ (29/30 and 12/13, respectively). Only one study found *TERTp* mutations in FA:^31^ 4 cases out of 58 (7%) and all of the *TERTp*^*Mut+*^ FA also harboured *MED12* mutations. Our study found no significant correlation between *MED12* and *TERTp* mutations, in either FA or PT. Although *MED12* mutations are most frequently observed in intracanalicular-type FA,^10,11,21,23,24,28^ *TERTp* mutations detected in our study had no statistical relationship with FA classification or PT grade (Table 3). These two mutations might independently affect the genesis or development of FA and PT.

Among the 72 patients in our study, three experienced recurrences (Table 4). When we compared the first resected tumour with later ones, two of them, an FA and a benign PT, showed *TERTp*^*Mut*^ status change from negative to positive. Considering that all cases harboured the same pattern of *MED12* mutations within each case, the secondary resected tumours seemed to be genetically identical to the primary tumours, suggesting that they were truly recurrent tumours. Garcia-Dios et al. also reported evidence of recurrent PT that acquired *TERTp* mutations,^33^ but our result indicates that *TERTp* mutations can be acquired during tumour growth or recurrence in both FA and PT.

We detected *TERTp* mutations at a much higher rate in FA than were seen in previous studies,^29,31,33^ but some might doubt the accuracy of the FA diagnoses. In the current study, two expert pathologists diagnosed all of the tumours. The distinction between FA and PT is often challenging, and in fact, we had eight cases, all eventually diagnosed as benign PT, on which the pathologists did not initially agree whether they were intracanalicular-type FA or benign PT (data not shown). *TERTp* mutations were found in five of these controversial tumours (63%), which is similar to the mutation rate of PT as a whole (61%), but much higher than that of FA (30%, Table 3). We consider that these controversial cases were credibly distinguished from other FA, and would be similarly diagnosed by most pathologists.

Although we found ddPCR to be more sensitive than SS for *TERTp* mutations, technical challenges remain. Sufficient quality and quantity of DNA is essential for detecting low-frequency mutations; therefore, small tumours or hyalinised FA may be inadequate for mutation analysis with ddPCR. Another challenge to successful analysis is artefacts from formalin-fixed samples when using sensitive molecular assays causing false-positive calls.^52,53^ We set thresholds based on signals from empty droplets, wild-type DNA and mutant-positive controls, and determined samples as negative when they showed fewer than ten mutation calls, to decrease the possibility of error due to false positives. In other words, detecting very low-frequency mutations with fewer than ten calls is highly challenging. Additional studies are required to resolve these problems and improve accuracy.

To examine the influence of FFPE-derived artefacts of DNA in ddPCR analysis, it would be more robust to prepare an FFPE-derived negative and a positive control for both C228T and C250T mutation-positive controls. We set distilled water as a negative control, and used DNA collected from a cell line as a positive control, so the DNA of these controls did not derive from FFPE. However, in our positive control, only C228T positivity was confirmed; thus, it did not show what type of dot plot would be obtained by ddPCR when C250T had a mutation in our study, although this was depicted in previous reports. These matters can be considered limitations of this work.

Another limitation is the primers that we used in this study, which were the same as those reported by Yoshida et al.^31^ and Liu et al.^34^ The nucleotide sequences of these primers differ from those in the study by McEvoy et al.,^39^ albeit by only a few bases. As there is no previous report describing the performance of ddPCR analysis using our primers, it may have been necessary to verify the findings of the study by using the primers that McEvoy et al. used for validation.

Extracted tumour DNA in the present study contained mixtures of that from epithelium, stroma or somewhat normal mammary cells and lymphocytes surrounding the tumour. Earlier studies found mutations for *TERTp* and *MED12* only in PT stromal components,^29,31,32^ so strictly speaking, DNA extraction from only the stromal components through microdissection would be necessary to identify *TERTp* mutations in FA and PT stroma, and to measure the allele frequency by ddPCR. Extracting DNA and measuring *TERTp* mutations from only the tumour’s stromal components may solve the problem of false positivity and -negativity more precisely, thus enhancing studies of relationships of *TERTp*^*Mut*^ allelic frequency with tumour growth rate and malignancy in FA and PT. Further study with targeted tumour cells is warranted.

In conclusion, we have presented the first assessment by ddPCR of *TERTp* mutations in FFPE breast FA and PT, and detected these mutations at a higher rate than previously reported. Our new findings reconfirm the genomic similarity of FA to PT and may help elucidate the biology of these tumours. We have shown ddPCR to be a robust method of detecting *TERTp* mutations, which suggest a wider clinical potential for this technology. A large-scale study is needed to determine whether *TERTp* mutation detection by ddPCR can be predictive, and has prognostic value for the surgical treatment of FA and PT.

## Supplementary information

Supplementary Information

## Data Availability

All data supporting the study are available on request. No proprietary materials, except patient tissues, were used.

## References

[1] Tavassoli, F. A. & Eusebi, V. Biphasic tumors. in *AFIP Atlas of Tumor Pathology*, Vol 10 (eds Tavassoli, F. A & Eusebi, V.) 315–340 (American Registry of Pathology, Washington, DC, 2009).

[2] Tan, P. H., Tse, G., Lee, A., Simpson, J. F. & Hanby, A. M. Fibroepithelial tumours. in *WHO Classification of Tumours of the Breast*, 4th edn. (eds Lakhani, S. R., Ellis, I. O., Schnitt, S. J., Tan, P. H. & van de Vijver, M. J.) 142–147 (IARC Press, Lyon, France, 2012).

[3] Yang X, Kandil D, Cosar EF, Khan A (2014). Fibroepithelial tumors of the breast: pathologic and immunohistochemical features and molecular mechanisms. Arch. Pathol. Lab. Med..

[4] Tan PH, Ellis IO (2012). Myoepithelial and epithelial-myoepithelial, mesenchymal and fibroepithelial breast lesions: updates from the WHO Classification of Tumours of the Breast. J. Clin. Pathol..

[5] Brogi, E. Fibroepithelial neoplasms. in *Rosen’s Breast Pathology*, 4th edn. (eds Hoda, S. A., Brogi, E., Koerner, F. C. & Rosen, P. R.) 213–270 (Lippincott Williams and Willkins, Philadelphia, PA, 2009).

[6] Sapino A, Bosco M, Cassoni P, Castellano I, Arisio R, Cserni G (2006). Estrogen receptor-beta is expressed in stromal cells of fibroadenoma and phyllodes tumors of the breast. Mod. Pathol..

[7] Tan WJ, Chan JY, Thike AA, Lim JC, Md Nasir ND, Tan JS (2016). MED12 protein expression in breast fibroepithelial lesions: correlation with mutation status and oestrogen receptor expression. J. Clin. Pathol..

[8] Carty NJ, Carter C, Rubin C, Ravichandran D, Royle GT, Taylor I (1995). Management of fibroadenoma of the breast. Ann. R. Coll. Surg. Engl..

[9] Dixon JM (1991). Cystic disease and fibroadenoma of the breast: natural history and relation to breast cancer risk. Br. Med. Bull..

[10] Mishima C, Kagara N, Tanei T, Naoi Y, Shimoda A, Shimazu K (2015). Mutational analysis of MED12 in fibroadenomas and phyllodes tumors of the breast by means of targeted next-generation sequencing. Breast Cancer Res. Treat..

[11] Yoshida M, Sekine S, Ogawa R, Yoshida H, Maeshima A, Kanai Y (2015). Frequent MED12 mutations in phyllodes tumours of the breast. Br. J. Cancer.

[12] Cani AK, Hovelson DH, McDaniel AS, Sadis S, Haller MJ, Yadati V (2015). Next-Gen sequencing exposes frequent MED12 mutations and actionable therapeutic targets in phyllodes tumors. Mol. Cancer Res..

[13] Nagasawa S, Maeda I, Fukuda T, Wu W, Hayami R, Kojima Y (2015). MED12 exon 2 mutations in phyllodes tumors of the breast. Cancer Med..

[14] Ben Hassouna J, Damak T, Gamoudi A, Chargui R, Khomsi F, Mahjoub S (2016). Phyllodes tumors of the breast: a case series of 106 patients. Am. J. Surg..

[15] Guerrero MA, Ballard BR, Grau AM (2003). Malignant phyllodes tumor of the breast: review of the literature and case report of stromal overgrowth. Surg. Oncol..

[16] Noguchi S, Yokouchi H, Aihara T, Motomura K, Inaji H, Imaoka S (1995). Progression of fibroadenoma to phyllodes tumor demonstrated by clonal analysis. Cancer.

[17] Noguchi S, Aihara T, Motomura K, Inaji H, Imaoka S, Koyama H (1996). Phyllodes tumor of the breast: pathology, genesis, diagnosis, and treatment. Breast Cancer.

[18] Millikan R, Hulka B, Thor A, Zhang Y, Edgerton S, Zhang X (1995). p53 mutations in benign breast tissue. J. Clin. Oncol..

[19] Franco N, Picard SF, Mege F, Arnould L, Lizard-Nacol S (2001). Absence of genetic abnormalities in fibroadenomas of the breast determined at p53 gene mutations and microsatellite alterations. Cancer Res..

[20] Vorkas PA, Poumpouridou N, Agelaki S, Kroupis C, Georgoulias V, Lianidou ES (2010). PIK3CA hotspot mutation scanning by a novel and highly sensitive high-resolution small amplicon melting analysis method. J. Mol. Diagn..

[21] Loke BN, Md Nasir ND, Thike AA, Lee JYH, Lee CS, The BT (2018). Genetics and genomics of breast fibroadenomas. J. Clin. Pathol..

[22] Lim WK, Ong CK, Tan J, Thike AA, Ng CC, Rajasegaran V (2014). Exome sequencing identifies highly recurrent MED12 somatic mutations in breast fibroadenoma. Nat. Genet..

[23] Pfarr N, Kriegsmann M, Sinn P, Klauchen F, Endris V, Herpel E (2015). Distribution of MED12 mutations in fibroadenomas and phyllodes tumors of the breast–implications for tumor biology and pathological diagnosis. Genes Chromosomes Cancer.

[24] Piscuoglio S, Murray M, Fusco N, Marchiò C, Loo FL, Martelotto LG (2015). MED12 somatic mutations in fibroadenomas and phyllodes tumours of the breast. Histopathology.

[25] Lien HC, Huang CS, Yang YW, Jeng YM (2016). Mutational analysis of *MED12* exon 2 in a spectrum of fibroepithelial tumours of the breast: implications for pathogenesis and histogenesis. Histopathology.

[26] Ng CC, Tan J, Ong CK, Lim WK, Rajasegaran V, Nasir ND (2015). MED12 is frequently mutated in breast phyllodes tumours: a study of 112 cases. J. Clin. Pathol..

[27] Tan J, Ong CK, Lim WK, Ng CC, Thike AA, Ng LM (2015). Genomic landscapes of breast fibroepithelial tumors. Nat. Genet..

[28] Kim JY, Yu JH, Nam SJ, Kim SW, Lee SK, Park WY (2018). Genetic and clinical characteristics of phyllodes tumors of the breast. Transl. Oncol..

[29] Piscuoglio S, Ng CK, Murray M, Burke KA, Edelweiss M, Geyer FC (2016). Massively parallel sequencing of phyllodes tumours of the breast reveals actionable mutations, and TERT promoter hotspot mutations and TERT gene amplification as likely drivers of progression. J. Pathol..

[30] Pareja F, Geyer FC, Kumar R, Selenica P, Piscuoglio S, Ng CKY (2017). Phyllodes tumors with and without fibroadenoma-like areas display distinct genomic features and may evolve through distinct pathways. NPJ Breast Cancer.

[31] Yoshida M, Ogawa R, Yoshida H, Maeshima A, Kanai Y, Kinoshita T (2015). TERT promoter mutations are frequent and show association with MED12 mutations in phyllodes tumors of the breast. Br. J. Cancer.

[32] Tsang JYS, Hui YK, Lee MA, Lacambra M, Ni YB, Cheung SY (2018). Association of clinicopathological features and prognosis of TERT alterations in phyllodes tumor of breast. Sci. Rep..

[33] Garcia-Dios DA, Levi D, Shah V, Gillett C, Simpson MA, Hanby A (2018). MED12, TERT promoter and RBM15 mutations in primary and recurrent phyllodes tumours. Br. J. Cancer.

[34] Liu SY, Joseph NM, Ravindranathan A, Stohr BA, Greenland NY, Vohra P (2016). Genomic profiling of malignant phyllodes tumors reveals aberrations in FGFR1 and PI-3 kinase/RAS signaling pathways and provides insights into intratumoral heterogeneity. Mod. Pathol..

[35] Clark AD, Oldenbroek M, Boyer TG (2015). Mediator kinase module and human tumorigenesis. Crit. Rev. Biochem. Mol. Biol..

[36] Yuan P, Cao JL, Abuduwufuer A, Wang LM, Yuan XS, Lv W (2016). Clinical characteristics and prognostic significance of TERT promoter mutations in cancer: a cohort study and a meta-analysis. PLoS ONE.

[37] Vinagre J, Almeida A, Pópulo H, Batista R, Lyra J, Pinto V (2013). Frequency of TERT promoter mutations in human cancers. Nat. Commun..

[38] McEvoy AC, Calapre L, Pereira MR, Giardina T, Robinson C, Khattak MA (2017). Sensitive droplet digital PCR method for detection of TERT promoter mutations in cell free DNA from patients with metastatic melanoma. Oncotarget.

[39] McEvoy AC, Wood BA, Ardakani NM, Pereira MR, Pearce R, Cowell L (2018). droplet digital PCR for mutation detection in formalin-fixed, paraffin-embedded melanoma tissues: a comparison with Sanger sequencing and pyrosequencing. J. Mol. Diagn..

[40] Vogelstein B, Kinzler KW (1999). Digital PCR. Proc. Natl Acad. Sci. USA.

[41] Hindson BJ, Ness KD, Masquelier DA, Belgrader P, Heredia NJ, Makarewicz AJ (2011). High-throughput droplet digital PCR system for absolute quantitation of DNA copy number. Anal. Chem..

[42] Taylor SC, Carbonneau J, Shelton DN, Boivin G (2015). Optimization of droplet digital PCR from RNA and DNA extracts with direct comparison to RT-qPCR: clinical implications for quantification of oseltamivir-resistant subpopulations. J. Virol. Methods.

[43] Kuroda H, Takeuchi I, Ohnishi K, Sakamoto G, Akiyama F, Toyozumi Y (2006). Clinical and pathologic features of fibroadenoma of the mastopathic type. Surg. Today.

[44] Mori I, Han B, Wang X, Taniguchi E, Nakamura M, Nakamura Y (2006). Mastopathic fibroadenoma of the breast: a pitfall of aspiration cytology. Cytopathology.

[45] Sklair-Levy M, Sella T, Alweiss T, Craciun I, Libson E, Mally B (2008). Incidence and management of complex fibroadenomas. Am. J. Roentgenol..

[46] Wechselberger G, Schoeller T, Piza-Katzer H (2002). Juvenile fibroadenoma of the breast. Surgery.

[47] Je EM, Kim MR, Min KO, Yoo NJ, Lee SH (2012). Mutational analysis of MED12 exon 2 in uterine leiomyoma and other common tumors. Int. J. Cancer.

[48] Forbes SA, Beare D, Gunasekaran P, Leung K, Bindal N, Boutselakis H (2015). COSMIC: exploring the world’s knowledge of somatic mutations in human cancer. Nucleic Acids Res.

[49] Corless BC, Chang GA, Cooper S, Syeda MM, Shao Y, Iman Osman I (2019). Development of novel mutation-specific droplet digital PCR assays detecting TERT promoter mutations in tumor and plasma samples. J. Mol. Diagn..

[50] Sho S, Court CM, Kim S, Braxton DR, Hou S, Muthusamy VR (2017). Digital PCR improves mutation analysis in pancreas fine needle aspiration biopsy specimens. PLoS ONE.

[51] Nault JC, Calderaro J, Di Tommaso L, Balabaud C, Zafrani ES, Bioulac-Sage P (2014). Telomerase reverse transcriptase promoter mutation is an early somatic genetic alteration in the transformation of premalignant nodules in hepatocellular carcinoma on cirrhosis. Hepatology.

[52] Ye X, Zhu ZZ, Zhong L, Lu Y, Sun Y, Yin X (2013). High T790M detection rate in TKI-naive NSCLC with EGFR sensitive mutation: truth or artifact?. J. Thorac. Oncol..

[53] Watanabe M, Kawaguchi T, Isa S, Ando M, Tamiya A, Kubo A (2015). Ultra-sensitive detection of the pretreatment EGFR T790M mutation in non-small cell lung cancer patients with an EGFR-activating mutation using droplet digital PCR. Clin. Cancer Res..

