# Supplementary material for: Droplet-digital PCR reveals frequent mutations in TERT promoter region in breast fibroadenomas and phyllodes tumours, irrespective of the presence of MED12 mutations
Source: Br J Cancer. 2020 Oct 13;124(2):466–73. doi: 10.1038/s41416-020-01109-8 (PMC7852881; doi:10.1038/s41416-020-01109-8)
Supplement: Supplementary file 1 — Supplementary Information [file 41416_2020_1109_MOESM1_ESM.pdf]

Droplet-digital PCR reveals frequent mutations in *TERT* promoter region in breast fibroadenoma and phyllodes tumours, irrespective of the presence of *MED12* mutations

Supplemental Figure

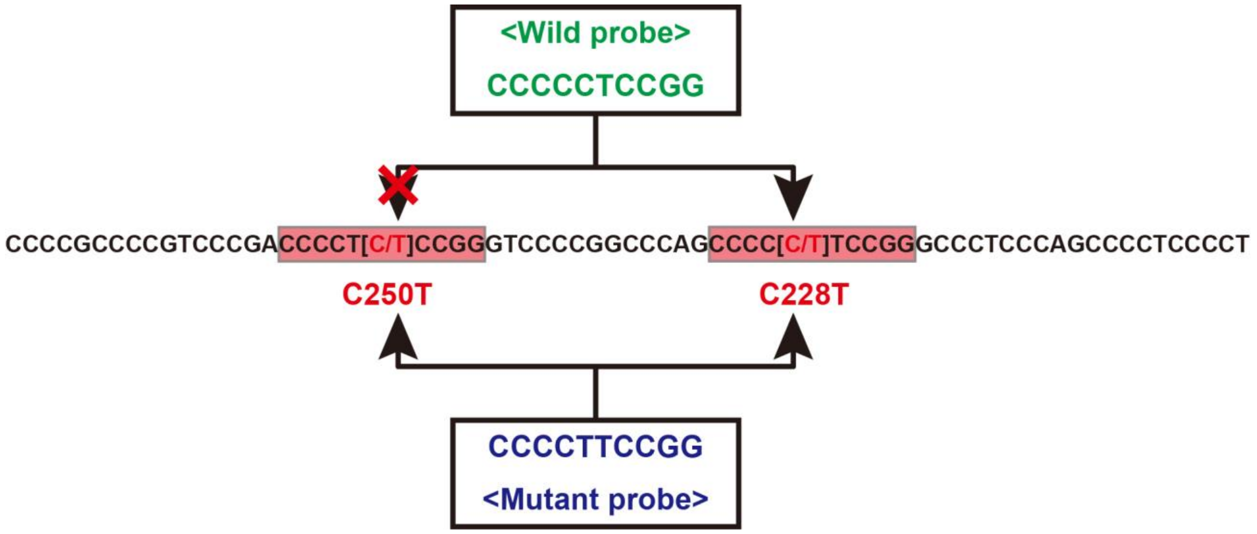

Figure S1. Locations of Sanger sequencing and ddPCR assay probes for to identifying C228T and C250T *TERT* promoter mutations

Probes to identify wild-type and mutant sequences are indicated. C228T and C250T are detected by the same mutant- probe, whereas the wild-type probe binds to only one site.

Supplemental Tables

Table S1. Forward and reverse primers used for Sanger sequencing and droplet digital PCR

Gene	Exon/region	Forward primer	Reverse primer	Reference
MED12	2	5′-AACTAAACGCCGCTTTCCTG-3′	5′-TTCCTTCAGCCTGGCAGAG-3′	10, 47
TERT	Promoter	5′-AGCGCTGCCTGAAACTCG-3′	5′-CCTGCCCCTTCACCTTCCAG-3′	31, 34

Table S2. Relationship between *MED12* mutations and histological characteristics of fibroadenomas and phyllodes tumors

Histology		Tumors, *n*	Tumors with *MED12* mutation, *n* (%)	*P*
Total		75	40 (53%)	
FA		44	19 (42%)	0.035
PT		31	21 (68%)	
FA	Intracanalicular	18	11 (58%)	0.046
	Non-intracanalicular	26	8 (31%)	
	Pericanalicular	6	1 (17%)	
	Mastopathic	8	4 (50%)	
	Organoid	7	1 (14%)	
	Complex	4	1 (25%)	
	Juvenile	1	1 (100%)	
PT	Benign	17	11 (65%)	0.81
	Borderline	9	6 (67%)	
	Malignant	5	4 (80%)	

FA fibroadenoma, *PT* phyllodes tumor

Table S3. Cases analyzed for *TERTp* mutation by both Sanger sequence and ddPCR

ID#	Age (year old)	Diagnosis	FA classification or PT grade	Tumor size (mm)	*MED12* mutation	*TERTp* mutation by Sanger sequence	*TERTp* mutation by ddPCR
15a	64	PT	Malignant	30	**c.130G>A**	Wild	Wild
15b	30	FA	Intracanalicular	23	Wild	Wild	Wild
15c	29	FA	Intracanalicular	40	**c.131G>A**	Wild	Wild
15d	33	FA	Intracanalicular	30	**c.130G>T**	Wild	Wild
15e	42	PT	Borderline	80	**c.131G>T**	Wild	**C228T**
15f	41	PT	Benign	23	Wild	Wild	Wild
16a	42	PT	Borderline	73	**c.130G>A**	**C228T**	**C228T**
16b	19	FA	Juvenile	52	**c.122-154del33**	Wild	**C228T**
16c	46	FA	Intracanalicular	25	**c.130G>A**	Wild	Wild
16d	62	PT	Benign	52	Wild	Wild	**C228T**
16e	60	FA	Intracanalicular	5	Wild	Wild	Wild
16f	24	FA	Intracanalicular	64	Wild	Wild	**C228T**
16g	21	PT	Benign	50	Wild	Wild	Wild
16h	29	FA	Intracanalicular	40	Wild	Wild	Wild
16i	50	PT	Benign	48	**c.130G>T**	**C228T**	**C228T**
16j	18	FA	Intracanalicular	23	**c.130G>A**	Wild	Wild
16k	21	FA	Intracanalicular	73	Wild	Wild	**C228T**

FA fibroadenoma, *PT* phyllodes tumor

Table S4. ***MED12*** **mutation,** ***TERT*** **promoter mutation and fractional abundance in FA and PT**

	Case	*MED12* mutation	*TERTp* mutation	Fractional abundance (%)
FA	15b	Negative	Negative	13
	15c	Positive	Negative	14
	15d	Positive	Negative	11
	16j	Positive	Negative	22
	16k	Negative	Positive	2.5
	16b	Positive	Positive	3.9
	16c	Positive	Negative	8.0
	16e	Negative	Negative	29
	16f	Negative	Positive	5.0
	16h	Negative	Negative	1.7
	A-1	Negative	Negative	19
	A-10	Positive	Negative	12
	A-11	Negative	Positive	31
	A-12	Negative	Negative	89
	A-13	Negative	Negative	3.3
	A-14	Negative	Negative	1.0
	A-2	Positive	Negative	11
	A-6	Negative	Negative	18
	A-7	Negative	Negative	75
	A-8	Negative	Negative	35
	A-9	Negative	Negative	14
	B-1	Positive	Negative	60
	B-10	Negative	Negative	23
	B-11	Positive	Positive	66
	B-12	Negative	Negative	3.5
	B-14	Positive	Positive	5.7
	B-16	Positive	Negative	9.0
	B-18	Negative	Positive	2.5
	B-19	Negative	Positive	8.4

	B-3	Negative	Positive	11
	B-4	Negative	Positive	23
	B-5	Positive	Positive	65
	B-6	Negative	Negative	17
	B-7	Positive	Negative	16
	B-8	Positive	Negative	35
	B-9	Negative	Positive	4.0
	C-1	Negative	Negative	16
	C-2	Positive	Negative	23
	C-4	Positive	Negative	N/A
	C-5	Positive	Negative	N/A
	C-6	Positive	Positive	14
	C-7	Negative	Negative	6.0
	C-8	Negative	Negative	5.7
	C-9	Positive	Negative	N/A
PT	12	Positive	Positive	9.0
	D-7	Positive	Positive	92
	5	Positive	Positive	34
	D-4	Positive	Positive	28
	16i	Positive	Positive	28.8
	D-6	Positive	Positive	52
	D-9	Positive	Positive	13
	D-8	Positive	Positive	15
	11	Negative	Positive	64
	16d	Negative	Positive	3.3
	8	Positive	Negative	42
	D-2	Positive	Negative	N/A
	D-5	Positive	Negative	90
	1	Negative	Negative	10
	15f	Negative	Negative	0.7
	D-3	Negative	Negative	20
	16g	Negative	Negative	2.7

16a	Positive	Positive	52.3
9	Positive	Positive	27.9
2	Positive	Positive	60
15	Positive	Positive	N/A
15e	Positive	Positive	5.3
16	Negative	Positive	76
14	Positive	Negative	3.8
3	Negative	Negative	70
7	Negative	Negative	2.4
6	Positive	Positive	87
13	Positive	Positive	84.8
10	Negative	Positive	87
15a	Positive	Negative	2.2
4	Positive	Negative	86

FA fibroadenoma, *PT* phyllodes tumor

Table S5. Fractional abundance in cases analysed with both SS and ddPCR for *TERT* promoter mutation detection

		TERTp mutation status		Fractional Abundance (%)
		Sanger sequencing	ddPCR	
FA	15b	Negative	Negative	13
	15c	Negative	Negative	14
	15d	Negative	Negative	11
	16b	Negative	Positive	3.9
	16c	Negative	Negative	8
	16e	Negative	Negative	29
	16f	Negative	Positive	5
	16h	Negative	Negative	1.7
	16j	Negative	Negative	22
	16k	Negative	Positive	2.5
PT	15a	Negative	Negative	2.2
	15e	Negative	Positive	5.3
	15f	Negative	Negative	0.7
	16a	Positive	Positive	52.3
	16d	Negative	Positive	3.3
	16g	Negative	Negative	2.7
	16i	Positive	Positive	28.8

FA fibroadenoma, *PT* phyllodes tumor
